# Monthly Summary

**Published:** 1891-04

**Authors:** 


### Monthly Summary.

THE PHYSIOLOGICAL ACTION OF SULPHALDEHYDE.—Sulphaldehyde, produced by the action of sulphuretted hydrogen on ethylic aldehyde, occurs in the form of an oleaginous liquid of a repulsive odor, solidifying slightly below the freezing-point. Treated with acids, sulphaldehyde is transformed into a solid, the point of fusion of the various articles of commerce varying in accordance with their greater or less purity. Its analogy in composition to paraldehyde led Dr. Lusini to test the physiological action of the sulphaldehyde, administering it by injections intraperitoneally, and into the stomach of rabbits and frogs (*Les Nouveaux Remedes*, November 8, 1800).

It appears that sulphaldehyde produces profound and and tranquil sleep without any phenomena of excitation. On account of its insolubility, some time is required before its effects follow its administration; nevertheless, it is claimed to be much more energetic than paraldehyde. It is eliminated completely by the urine, to which it gives its characteristic odor.—*Therapeutic Gazette.*

